# Editorial for the Special Issue “Antibacterial Activity of Drug-Resistant Strains”

**DOI:** 10.3390/ijms25031878

**Published:** 2024-02-04

**Authors:** Marisa Di Pietro, Simone Filardo, Rosa Sessa

**Affiliations:** Department of Public Health and Infectious Diseases, Sapienza University, P.le Aldo Moro, 5, 00185 Rome, Italy; marisa.dipietro@uniroma1.it (M.D.P.); rosa.sessa@uniroma1.it (R.S.)

Antimicrobial resistance is an urgent global public health threat, as approximately 700,000 deaths annually can be attributed to antibiotic-resistant bacterial infections, and this figure is expected to reach 10 million deaths/year by 2050, a number that greatly exceeds the number of deaths resulting from cancer [1,2,3].

The widespread use of antimicrobials in clinical and community settings, as well as in livestock and crop production, is considered one of the main drivers of the phenomenon of antimicrobial resistance [4,5,6]. As a results, over the years, an increased incidence of bacterial resistance has been observed, beginning with the emergence of methicillin-resistant *Staphylococcus aureus*, which has rapidly become the most frequently occurring resistant pathogen, with it being identified in many parts of the world, including Europe [7,8]. A more recent issue is the increasing prevalence of extended-spectrum beta-lactamase (ESBL)-producing *Enterobacteriaceae* all over the world, further limiting treatment options [9,10]. Among the pathogens with growing multidrug resistance, the WHO has included the ESKAPE pathogens (*Enterococcus faecium*, *Staphylococcus aureus*, *Klebsiella pneumoniae*, *Acinetobacter baumannii*, *Pseudomonas aeruginosa*, and *Enterobacter* species), against which new antibiotics are urgently needed [11,12,13].

In 2015, the WHO developed an action plan to combat antimicrobial resistance, and this plan includes several strategic objectives, such as to improve the awareness and understanding of antimicrobial resistance; to strengthen knowledge and evidence-based guidelines through surveillance and research; to optimize the use of antimicrobials in humans and animals; and to develop the economic case for sustainable investment in new medicines and other interventions to meet the needs of all countries [14,15].

Against this backdrop, this Special Issue includes original articles and reviews that provide insights into antibiotic resistance as well as the development of strategies to counter it, such as surveillance studies of bacterial strains, which may help to alleviate public health concerns, and the search for alternative compounds as potential antimicrobial agents. This Editorial highlights the key findings derived from the published manuscripts.

Mirabela Romanescu’s article (contribution 1) is a systematic review that describes antimicrobial resistance and its main determinants, the modality by which the issue has been globally addressed, and the potential of essential oils as an alternative or auxiliary therapy option. The authors focused on the pathogenesis, mechanisms of resistance, and activities of several essential oils against the six high priority pathogens listed by WHO in 2017 [16]. Lastly, the authors evidenced the need for standardized techniques for analyzing the antimicrobial activities of essential oils, given the heterogeneity of the research designs and techniques used in different studies.

The article by Yin-Chao Tong et al. (contribution 2) is a prevalence study on feline-derived ESBL *Escherichia coli* and their resistance to antibiotics that considers the potential risk of transmission of this pathogen to humans, especially between pets and their owners; this study investigated the presence of ESBL genes in feline-derived *E. coli* strains in different areas of China.

Jurgita Aksomaitiene’s article (contribution 3) provides valuable insights into the mechanisms of antimicrobial resistance and the genetic relatedness of *Campylobacter jejuni* strains isolated from broiler products, cattle, wild birds, and human feces via whole-genome sequencing and core-genome MLST techniques. In particular, the authors revealed a potential transmission route for *C. jejuni* between humans and animals, alongside the presence of multiple gene mutations responsible for antimicrobial resistance in *C. jejuni* genomes, suggesting that Campylobacters in Lithuania have been exposed to selective pressure, mainly in the form of antimicrobial use.

The article by Sattaporn Weawsiangsang et al. (contribution 4) demonstrates that hydroquinine, a compound derived from extracts of *Tetrigona apicalis*, had bacteriostatic and bactericidal activity against several human clinical isolates of *Pseudomonas aeruginosa*, including multidrug-resistant strains. Interestingly, molecular docking analysis and studies of gene expression patterns suggested arginine deiminase -pathway-related proteins as the potential molecular target of hydroquinine in eliciting its antimicrobial effects towards multidrug-resistant strains of *P. aeruginosa*.

The discovery of novel formulations for use as antibacterial agents is of great importance to fight not only *P. aeruginosa* [17,18,19], recognized as one of the greatest threats to human health, but also other emerging resistant pathogens, such as Chlamydiae [20,21,22,23,24,25]. In this regard, Marisa Di Pietro et al. (contribution 5) discovered that olive oil polyphenol-based formulations were effective against *C. trachomatis*, known as the leading cause of bacterial sexually transmitted diseases worldwide. In particular, the formulations were effective against chlamydial elementary bodies, responsible for the transmission and dissemination of the infection and, hence, for the development of chronic complications such as infertility.

Antimicrobial resistance has grown into a serious global menace that impacts not only humans but also animals and plants, and given this backdrop, Yin-Chao Tong’s article (contribution 2) is important, as it evidences the garlic oil’s ability to increase the susceptibility of feline-derived ESBL *E. coli* to cefquinome. Mizuki Kusumoto et al. (contribution 6) employed a pharmacokinetics–pharmacodynamics approach to establish appropriate treatment regimens for Flomoxef, an oxacephem antibiotic used in humans, against ESBL-producing Enterobacterales in dogs. This study demonstrated that dosage regimens of 40 mg/kg Flomex every 6 and 8 h can be a non-carbapenem treatment for canine infections of *Escherichia coli*, *Klebsiella pneumoniae*, and *Proteus mirabilis*, but not for *Enterobacter cloacae*. The article by JeongWoo Kang et al. (contribution 7) is a bioequivalence study on the generic tulathromycin products extensively utilized in veterinary medicine for treating respiratory infections in cattle and swine due to its broad-spectrum activity against a range of Gram-positive, Gram-negative, and atypical bacteria. This study demonstrates the bioequivalence of four generic tulathromycin products (Tulaject, Tulagen, Toulashot, and T-raxxin) compared to a reference product, Draxxin, using a statistical program for pharmacokinetic parameters. Lastly, Yue Zou’s article (contribution 8) systematically reviews the application of benzoxazole and benzothiazole derivatives in the discovery of new agrochemicals, summarizing their antibacterial, fungicidal, antiviral, herbicidal, and insecticidal activities. This paper discusses the structural–activity relationship and mechanism of action of these active compounds, aiming to provide new insights and inspiration for the discovery of new pesticides.

Overall, this Special Issue showcases a collection of relevant topics related to the One Health Approach to tackle the overlapping effects antimicrobial resistance is having on human and animal health, agriculture and food, and the environment.
